# Supplementary material for: Osteoclastogenic Potential of Tissue-Engineered Periosteal Sheet: Effects of Culture Media on the Ability to Recruit Osteoclast Precursors
Source: Int J Mol Sci. 2021 Feb 22;22(4):2169. doi: 10.3390/ijms22042169 (PMC7926432; doi:10.3390/ijms22042169)
Supplement: Supplementary file 1 [file ijms-22-02169-s001.pdf]

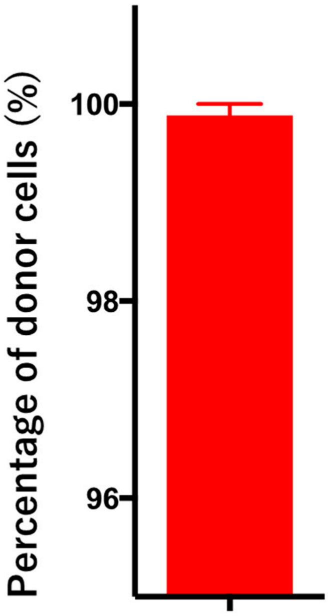

Figure S1. Donor chimerism at five weeks after BMT. Hematopoietic chimerism on blood was measured by Flow cytometric analysis five weeks post BMT. Anti-H-2b (AF6-88.5) and H-2d (SF1-1.1) antibodies were sourced by BioLegend (San Diego, CA, USA). Mean ± SD is shown. N = 6.

Table S1. Blood counts at five weeks after BMT.

White blood cells (per μL)	Hemoglobin (g/dL)	Platelets (per μL)
8376 ± 3397	16.2 ± 1.0	133.3 ± 21.5

Mean ± SD is shown. N = 21.
